# Comparison of the BCI Performance between the Semitransparent Face Pattern and the Traditional Face Pattern

**DOI:** 10.1155/2017/1323985

**Published:** 2017-04-09

**Authors:** Jiao Cheng, Jing Jin, Xingyu Wang

**Affiliations:** Key Laboratory of Advanced Control and Optimization for Chemical Processes, Ministry of Education, East China University of Science and Technology, Shanghai, China

## Abstract

Brain-computer interface (BCI) systems allow users to communicate with the external world by recognizing the brain activity without the assistance of the peripheral motor nervous system. P300-based BCI is one of the most common used BCI systems that can obtain high classification accuracy and information transfer rate (ITR). Face stimuli can result in large event-related potentials and improve the performance of P300-based BCI. However, previous studies on face stimuli focused mainly on the effect of various face types (i.e., face expression, face familiarity, and multifaces) on the BCI performance. Studies on the influence of face transparency differences are scarce. Therefore, we investigated the effect of semitransparent face pattern (STF-P) (the subject could see the target character when the stimuli were flashed) and traditional face pattern (F-P) (the subject could not see the target character when the stimuli were flashed) on the BCI performance from the transparency perspective. Results showed that STF-P obtained significantly higher classification accuracy and ITR than those of F-P (*p* < 0.05).

## 1. Introduction

Brain-computer interface (BCI) is a technology that allow users to communicate with others or control external devices via brain activity alone [1–3]. BCI directly measures brain activities usually based on electroencephalography (EEG) recorded noninvasively through electrodes placed on the surface of the head [4]. The intention of users can be recognized by analyzing the EEG signals of various mental tasks [5, 6] which can help these users directly control external devices through brain activities.

P300-based BCI is one of the most common used BCI systems presented by Farwell and Donchin [7], and this system uses the flash letter pattern. Over the past two decades, the “flash only” paradigm in which the target reverses color or is briefly masked by a solid box [8] is usually used as stimulus to elicit P300 potentials. However, recent studies are focused mostly on a new stimulus that the target is overlapped with a famous face. Such stimuli actually yield better performance compared with the conventional flash only pattern through numerous experiments [8–12]. This result is due to that the face stimuli can elicit other event-related potential (ERP) components not restricted to the P300 components (such as vertex positive potential (VPP), N170, N200, and N400), and these potentials also contribute to the classification accuracy. Zhang et al. [11, 12] reported that VPP and N170 can help improve classification accuracy with stimuli that change to faces. Jin et al. [8, 9, 13] also reported that N400 significantly contributes to improving the classification accuracy in ERP-based BCI system. Currently, a variety of face patterns have been proposed by numerous researchers. Jin et al. [8, 10] presented various types of face paradigms (neutral face, smiling face, shaking neutral face, and shaking smiling face paradigms) and compared multifaces using various familiar faces with single faces. The results indicated that the performance of the ERP-based BCI is enhanced by these face stimuli. Kaufmann et al. [9] introduced face stimuli transparently superimposed on characters in comparison with the flash pattern. Their result showed that such stimuli can generate higher ERP amplitudes and obtain higher classification accuracy than those of the flash letter pattern. However, previous studies on face stimuli focused mainly on the effect of various face types (i.e., face expression, face familiarity, and multifaces) on the BCI performance [8, 10, 14]. Studies on the influence of face transparency differences are scarce. Therefore, we investigated the effect of semitransparent face pattern (STF-P) (the subject could see the target character when the stimuli were flashed) and traditional face pattern (F-P) (the subject could not see the target character when the stimuli were flashed) on the BCI performance. Semitransparent faces can increase the psychological salience of the stimulus and allow for uninterrupted attention. Thus, we hypothesized that the persistent visible target character could help subjects focus on the target, increase N200 and N400 amplitudes, and improve the performance of ERP-based BCI using semitransparent face stimuli.

## 2. Methods

### 2.1. Subjects

Ten healthy subjects (2 females and 8 males, aged 22–26 years with the mean age of 23.5) participated in this study. The native language of the subjects is Mandarin Chinese. In addition, these subjects are all right-handed and have no known neurological disorders. They signed written consent form prior to the experiment, and the local ethics committee approved the consent form and experimental procedure before any subjects participated. Furthermore, all subjects had no previous BCI experiences.

### 2.2. Stimuli and Procedure

**Figure 1:**
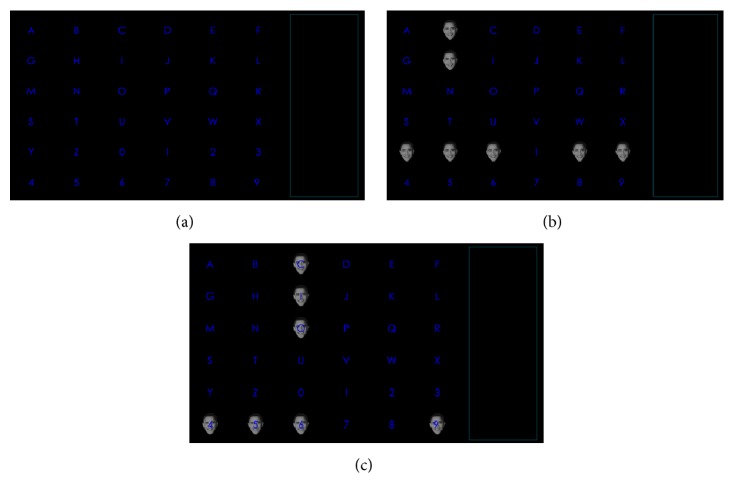
The display presented to subjects. (a) 6 × 6 matrix displayed in the monitor; (b) face pattern (F-P); (c) semitransparent face pattern (STF-P).

The subjects sat in a chair in front of the monitor, which displayed a 6 × 6 matrix with characters and numbers (Figure 1(a)) [10]. They were required to silently count the number of times the target flashed and avoid unnecessary movements. In this study, two paradigms were presented to the subjects. The parameters of the two paradigms (i.e., character size, intercharacter distance, background color, and stimulus brightness) were kept constant, except stimulus transparency. Accordingly, the counterbalance of the paradigm presentation could be obtained. In the first paradigm, the face stimulus concealed the target character, and the subject could not see the target character during the time the stimulus was on (Figure 1(b)). We called this paradigm as the traditional face pattern (F-P). In the second paradigm, the face stimulus was made semitransparent, and the subject could see the target character during the time the stimuli were on (Figure 1(c)). We called this paradigm as semitransparent F-P (STF-P). The subjects were tasked to count the number of times the target flashed. The flash configuration was based on binomial coefficients [15, 16]. The binomial coefficients were based on *C*(*n*, *k*) = *n*!/(*k*!(*n* − *k*)!), 0 ≤ *k* ≤ *n*, where *n* represents the number of flashes per trial and *k* represents the number of flashes per trial for each element in the matrix. We chose the combination of *C*(12,2) to denote the 12-flash pattern.  Table 1 shows the configuration of the 12-flash pattern combination with 36 flash pattern pairs. The positions in Table 1 corresponded to the positions of the 36 characters in a 6 × 6 matrix.

Each subject was required to complete two paradigms (F-P and STF-P) on the same day. Each paradigm contained one offline block and one online block. The order of the paradigms F-P and STF-P was counterbalanced throughout the experiment (five of ten subjects did the F-P first). During the offline period, each paradigm consisted of three offline runs called one offline block, and each offline run contained 5 target characters that would be spelled by the subjects without any rest. The subjects had 3 min rest between each offline run. In addition, each target character was identified through 16 trials, and each trial was composed of 12 flashes. No feedback would be presented to the subjects in the offline experiment. However, during the online period, each paradigm only had one online run called one online block. The number of trials for recognizing each target character was selected automatically by an adaptive strategy [17, 18], and each trial was also composed of 12 flashes. The subjects were required to spell 36 target characters without any rest during the online period, and the system would promptly present the online result whenever the classifier recognized the target character. The stimulus on time was 100 ms and the stimulus onset asynchrony was 250 ms throughout the offline and online experiments. Moreover, an italicized number was used to prompt the subjects of the next target character before each run started, and they had 4 s for target selection. In addition, after finishing the offline experiment, subjects had 4 min rest to prepare for the following online experiment. Copy spelling task was used in the offline and online experiments.

### 2.3. Calibration

**Figure 2:**
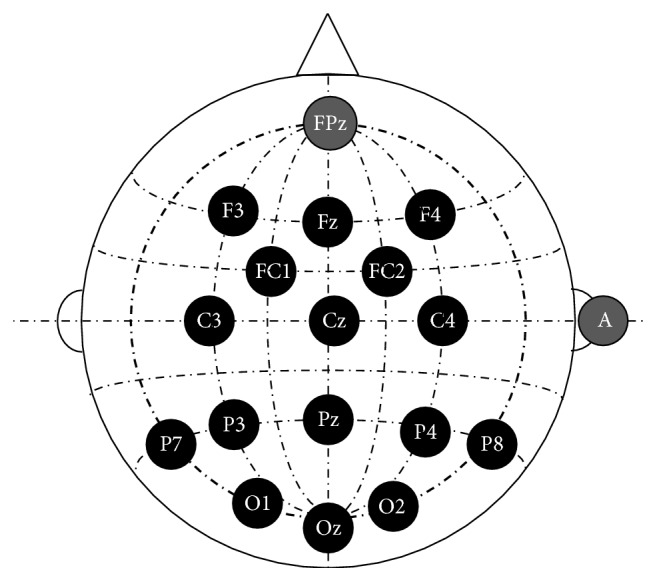
Configuration of the selected electrode positions (FPz was used as the ground electrode; right mastoid (A) was used as the reference electrode).

We acquired the EEG signals recorded with g.USBamp and g.EEGcap (Guger Technologies, Graz, Austria) with a sensitivity of 100 *μ*V, band-pass filtered between 0.5 and 30 Hz, and sampled at 256 Hz. A total of 16 corresponding electrode positions were selected in the experiments according to the International 10-20 System (Figure 2) [8]. These positions were Fz, FC1, FC2, C3, Cz, C4, P3, Pz, P4, O1, Oz, O2, P7, P8, F3, and F4. FPz was used as the ground electrode, while right mastoid (A) was used as the reference electrode. These selected electrodes were used to keep track of the EEG signals.

### 2.4. Feature Extraction Procedure

Feature extraction is an effective method for reducing dimensionality and amount of required computations [18]. In this study, the third-order Butterworth band-pass filter was used to filter the EEG signals; the high pass was 0.5 Hz and low pass was 30 Hz. In addition, we downsampled the EEG signals from 256 Hz to 36.6 Hz by selecting every seventh sample from the filtered EEG. Consequently, we obtained the feature vector with the size of 16 × 29 (16 represents the number of the channels and 29 denotes the time points). In addition, winsorizing was used to remove the electrooculogram interference signals. The 10th and 90th percentiles were computed for each sample. Amplitude values lying below the 10th percentile or above the 90th percentile were then replaced with the 10th percentile or the 90th percentile, respectively [19].

### 2.5. Classification Scheme

Bayesian linear discriminant analysis (BLDA) was used to build the classifier model for the online experiment. BLDA can effectively solve the problems of high-dimensional data sets or noise fitting owing to its regularization method. Hoffmann et al. [20] applied such a method to the classification of P300 BCIs and achieved good results.

### 2.6. Adaptive System Settings

The system was used to judge whether the results of two consecutive outputs were consistent. Accordingly, the final results of system output could be determined. If the results of two consecutive outputs were consistent, then the system exported the result as a feedback. Otherwise, the system would not provide any response until 16 trials were completed. When 16 trials ended, the classifier would automatically select the last output [21].

### 2.7. Statistical Analysis

We chose paired-samples *t*-tests (one-sample Kolmogorov-Smirnov test) for normal distribution to investigate the differences in mean amplitudes averaged from each ERP peak point ±20 ms between the F-P and STF-P paradigms. We also used such a method to explore the differences in online classification accuracy and bit rate between the two paradigms. The nonparametric Wilcoxon signed-rank test was used to compare the responses from the report of the subjects, as these data obey an uncertain distribution. The alpha level was *α* = 0.05.

### 2.8. Subjective Report

After finishing the tasks of two paradigms, we conducted a questionnaire survey of three questions. The three questions were answered by the subjects on a 1–3 scale. A high score indicated a high degree of tiredness, difficulty, and annoyance (1: minimum; 2: medium; 3: maximum). The questions were as follows:

1. Did this paradigm make you tired?
2. Was this paradigm difficult?
3. Did this paradigm make you annoyed?

## 3. Results

**Figure 3:**
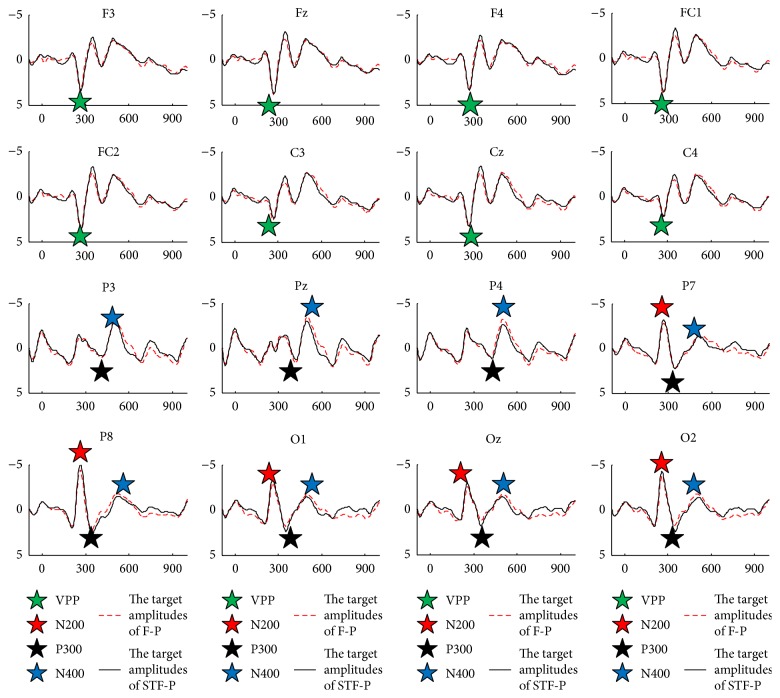
Grand averaged ERPs of target flashes across subjects 1–10 over 16 electrode sites.

**Figure 4:**
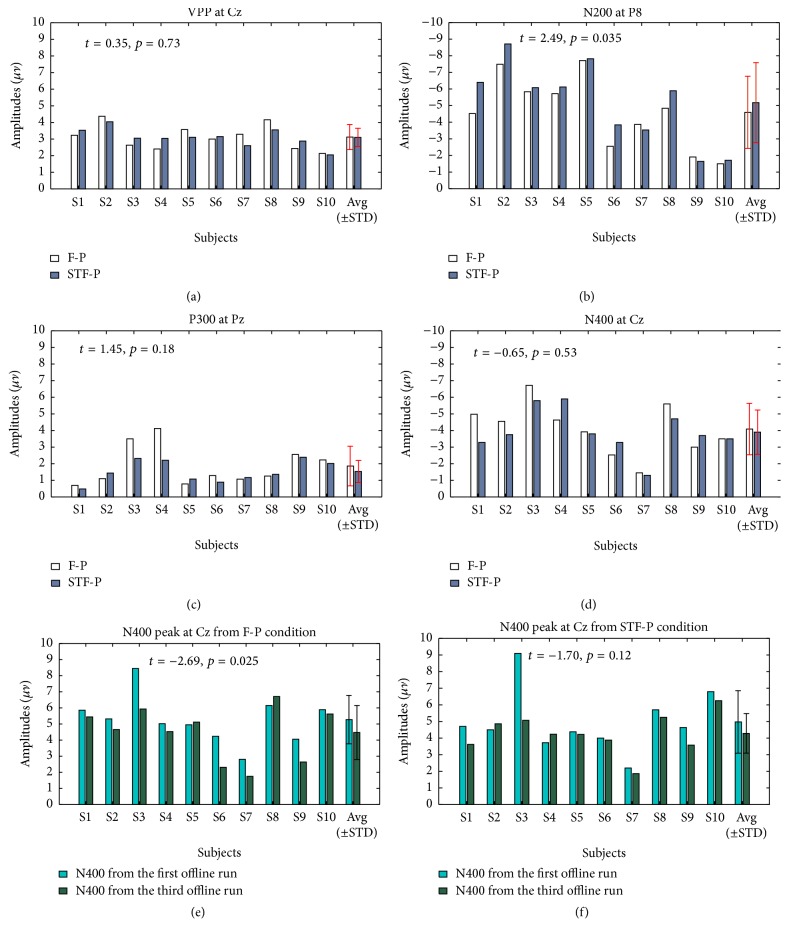
Six panels presenting the mean amplitudes averaged from each ERP peak point ±20 ms for each subject, and the differences in the amplitudes of N400 between the first and third offline runs from the two paradigms. These panels indicate the averaged amplitude of VPP at Cz (a); the averaged amplitude of N200 at P8 (b); the averaged amplitude of P300 at Pz (c); the averaged amplitude of N400 at Cz (d); the difference of N400 at Cz from the first and third offline run of the F-P (e); the difference of N400 at Cz from the first and third offline run of the STF-P (f). In addition, “Avg” is the average, “STD” is the standard deviation, and the error bars in the figure represent a standard deviation of each data set.

**Figure 5:**
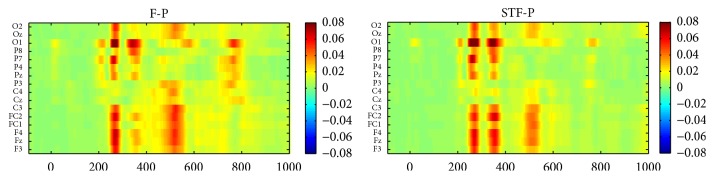
Absolute *R*-squared values of ERPs from these two patterns at 0–1000 ms averaged from subjects 1–10.

Figure 3 shows the grand averaged ERPs of target flashes after being baseline corrected by 100 ms prestimulus interval across subjects 1–10 over 16 electrode sites [21]. The two paradigms had similar VPP components over frontal and central areas. However, a few differences were found in N200 and P300 over parietal and occipital sites. STF-P had relatively higher peak values across N200 and P300 than those of F-P over parietal and occipital sites. We explored the differences of VPP, N200, P300, and N400 between STF-P and F-P; for this purpose, we selected Cz for VPP, P8 for N200, Pz for P300, and Cz for N400; these electrode positions typically contain the largest corresponding ERP components [7, 12, 22, 23] and are thus the best examples.  Figure 4 shows the mean amplitudes of VPP at Cz, N200 at P8, P300 at Pz, and N400 at Cz for each subject and the N400 amplitude of the first and the third runs at Cz from the two paradigms [8]. The mean amplitude was averaged from each ERP peak point ±20 ms. The N200 amplitude at P8 from STF-P was significantly larger than that of F-P (*t* = 2.49, *p* < 0.05, df = 9, Figure 4(b)). Furthermore, no significant difference was found between the two paradigms across VPP and P300 (*t* = 0.35, *p* > 0.05, df = 9 for VPP, and *t* = 1.45, *p* > 0.05, df = 9 for P300, Figures 4(a) and 4(c)). Meanwhile, the value of P300 was smaller than that of the P300 reported in other studies [19, 24]. Although the N400 at Cz from STF-P showed no significant difference compared with that from F-P (*t* = −0.65, *p* > 0.05, df = 9; Figure 4(d)), the stability of N400 from STF-P (*t* = −1.70, *p* > 0.05, df = 9; Figure 4(f)) was better than that from F-P (*t* = −2.69, *p* < 0.05, df = 9; Figure 4(e)).  Figure 5 shows the absolute *R*-squared values of ERPs from the two paradigms at 0–1000 ms averaged from subjects 1–10 on 16 electrodes. *R*-squared values of ERPs reflected the time energy of the signals. The definition is as follows:

(1) $$\begin{array}{c} r^{2}={\left(\;\frac{\sqrt{N_{1}N_{2}}}{N_{1}+N_{2}}\cdot \frac{mean\left(X_{1}\right)-mean\left(X_{2}\right)}{std\left(X_{1}\cup X_{2}\right)}\;\right)}^{2}, \end{array}$$

where *X*_1_ and *X*_2_ are the features of classes “1” and “2,” respectively and *N*_1_ and *N*_2_ are the number of samples.

**Figure 6:**
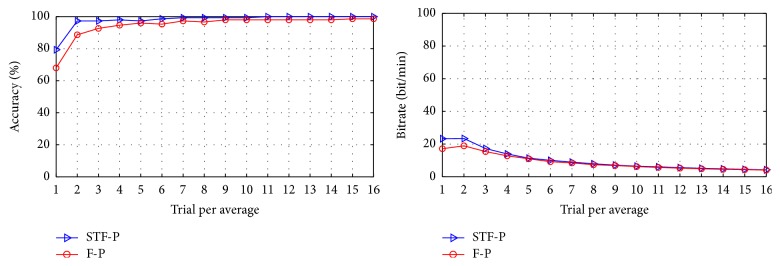
Classification accuracy and raw bit rate based on the offline data.

Figure 6 shows the classification accuracy and raw bit rate based on the offline data [8]. The values were obtained from 15-fold cross-validation. The classification accuracy and bit rate of STF-P were better than those of F-P when 1–16 numbers of trials were used for averaging.

**Figure 7:**
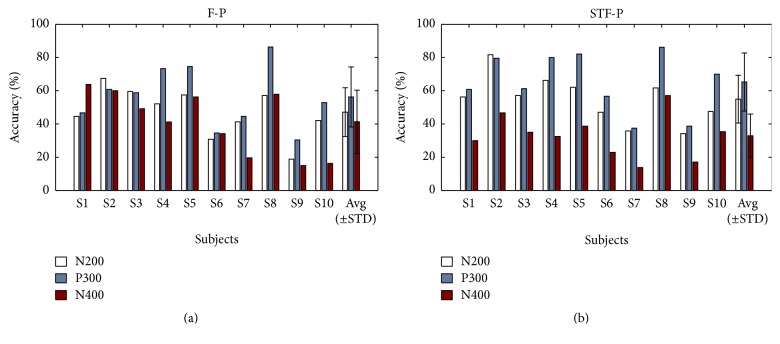
The contributions of N200, P300, and N400 time windows on the classification accuracy.

Figure 7 shows the contributions of N200 (between 150 and 300 ms), P300 (between 300 and 450 ms), and N400 (between 450 and 700 ms) on the BCI performance [8]. The two graphs indicated that N200 and P300 components were crucial in the classification accuracy. In addition, the N400 component also contributed to the classification accuracy.

Table 2 shows the online classification accuracy, bit rate, and mean number of trials for each subject. The classification accuracy and bit rate of STF-P were significantly higher than those of F-P (*t* = 2.89, *p* < 0.05, df = 9 for classification accuracy, *t* = 4.03, *p* < 0.05, df = 9 for bit rate). Moreover, the number of trials for averaging of STF-P was significantly less than that of F-P (*t* = −2.33, *p* < 0.05, df = 9).

Table 3 presents the responses of the subjects to the three questions for each paradigm. We further investigated the differences between the two paradigms. For this purpose, we chose the Wilcoxon signed-rank test method owing to the fact that the data satisfy an uncertain distribution. No significant differences were found between the two paradigms in terms of the degree of tiredness (*p* > 0.05), degree of difficulty (*p* > 0.05), and degree of annoyance (*p* > 0.05).

## 4. Discussion

This study aimed to survey whether any difference would be found between STF-P in which the subject could see the target character during the time the stimuli were on and F-P in which the target character was concealed during the time the stimuli were on. The results showed that the STF-P could elicit larger N200 component and improve the classification accuracy and bit rate of the BCI system compared with the F-P.

The Eriksen flanker task [25] is a commonly used experimental design to obtain N200 and is a kind of a mismatch paradigm [21]. In the present study, the STF-P elicited larger N200 component than the F-P. On the one hand, semitransparent face stimuli may lead to a high mismatch, thereby resulting in a large N200. On the other hand, the psychological salience of the stimuli can be exploited to elicit high ERP components not confined to the P300 components [9].

The difference of P300 amplitude between two paradigms in this paper was not clear and no significant difference was found. This phenomenon may be attributed to the low luminosity contrast when the background is black (the gray value of face was set 110, while the background was 255). However, low luminosity contrast leads to low visual fatigue. Li et al. [26] studied the effects of various luminosity contrasts on the BCI performance and found that low luminosity contrast produces small amplitude for P300 on average. A high luminosity contrast can result in bright, noticeable infrequent stimuli; as a result, subjects can easily concentrate their attention and efficiently identify the target characters. Therefore, low brightness may lead to increased effort or attention deployment in subjects. This finding may have an important implication for clinical application.

The effects of repetition can decrease the amplitude of N400, especially for long-term offline data recording [8]. In STF-P, given that the target differed, the stimulus also differed when the subject shifted their focus from one target to another. Since the significant difference of N400 between the first and third offline run was found in the F-P while not in the STF-P (see Figures 4(e) and 4(f)), it indicated that STF-P contained less repetition effects compared to the F-P.

Classification accuracy and ITR are the important indexes of BCI performance. In previous studies, face paradigms themselves have had good performances in accuracy (mean accuracy was higher than 90%) [8]. Therefore, even 1% increment in accuracy would be a good improvement. In this study, the averaged classification accuracy and bit rate of the STF-P were 95.0%, 42.6 bit/min, while those of the F-P were 91.9%, 38.0 bit/min, and were 3.1%, 4.6 bit/min, higher than those of the F-P. Figure 4(b) showed that the N200 at P8 of the STF-P was significantly higher than that of the F-P. Figure 7 showed that N200 could contribute to classification accuracy. Kaufmann et al. [27] reported that the potential of N200 can enhance the classification accuracy. Jin et al. [8] proved that other components can contribute to the classification accuracy under the condition of small P300 amplitudes. Table 2 showed that the classification accuracy and information transfer rate of the STF-P were significantly higher than that of the F-P (*p* < 0.05). As in all, the STF-P could obtain superior performance compared to the F-P.

This research studied two paradigms only (semitransparency and nontransparency) and focused less on the different transparent degrees based on the state of being transparent. However, this research provided a new idea on the studies of face stimuli and demonstrated that other distinct components could contribute strongly to the BCI performance.

## 5. Conclusion

In this study, we measured the performance of STF-P and F-P on BCI. The result indicated that STF-P was superior to F-P. In future studies, we will further verify the performance of the STF-P pattern on patients.

## Figures and Tables

**Table 1:** Configuration of the 12-flash pattern combination.

1,4	1,5	1,6	1,7	1,8	1,9
2,10	2,5	2,6	2,7	2,8	2,9
3,10	*3,11*	3,6	3,7	3,8	3,9
4,10	*4,11*	4,12	4,7	4,8	4,9
5,10	*5,11*	5,12	1,10	5,8	5,9
6,10	*6,11*	6,12	3,12	*2,11*	6,9

*Notes*. We named these 12-flash groups as “flash_1_, flash_2_,…, flash_12_.” The numbers in the table represent the target character of the 12 flashes. The same number in the configuration would simultaneously present stimuli. For example, letter “A” was flashed in flash_1_ and flash_4_. The italicized numbers represent the positions that simultaneously presented face stimuli during flash_11_.

**Table 2:** Online classification accuracy, bit rate, and average number of trials for each subject.

		S1	S2	S3	S4	S5	S6	S7	S8	S9	S10	AVG ± STD
ACC (%)	F-P	94.4	94.4	91.7	94.4	80.5	100	94.4	77.8	91.7	100	91.9 ± 7.3
	STF-P	97.2	94.4	97.2	100	88.9	97.2	97.2	83.3	94.4	100	95.0 ± 5.2
RBR (bit/min)	F-P	32.9	42.7	36.3	44.5	32.3	44.3	43.9	29.4	28.6	44.8	38.0 ± 6.7
	STF-P	41.0	44.5	43.0	50.3	39.3	43.0	42.5	36.1	36.6	49.6	42.6 ± 4.8
AVT	F-P	2.78	2.14	2.39	2.06	2.14	2.33	2.08	2.22	3.03	2.31	2.35 ± 0.32
	STF-P	2.36	2.06	2.25	2.06	2.08	2.25	2.28	2.03	2.50	2.08	2.20 ± 0.16

ACC = classification accuracy, RBR = raw bit rate (bit/min), AVT = average number of trials used to classify each character, STF-P = semitransparent face pattern, F-P = face pattern, AVG = average, and STD = standard deviation.

**Table 3:** Subjects' responses to three questions for each pattern.

		S1	S2	S3	S4	S5	S6	S7	S8	S9	S10	AVG ± STD
Tired	F-P	2	2	2	1	2	2	1	2	1	1	1.6 ± 0.52
	STF-P	1	2	1	2	1	1	1	1	1	1	1.2 ± 0.42
Difficult	F-P	2	1	1	2	1	1	2	2	1	2	1.5 ± 0.53
	STF-P	1	2	1	1	1	2	1	2	1	1	1.3 ± 0.48
Annoyed	F-P	1	2	1	2	1	1	1	2	1	1	1.3 ± 0.48
	STF-P	1	1	1	2	1	1	1	2	1	1	1.2 ± 0.42

F-P = face pattern; STF-P = semitransparent face pattern. AVG is average and STD is standard deviation.
